# Transcranial focused ultrasound targeting the default mode network for the treatment of depression

**DOI:** 10.3389/fpsyt.2025.1451828

**Published:** 2025-04-04

**Authors:** Jessica N. Schachtner, Jacob F. Dahill-Fuchel, Katja E. Allen, Christopher R. Bawiec, Peter J. Hollender, Sarah B. Ornellas, Soren D. Konecky, Achal S. Achrol, John J. B. Allen

**Affiliations:** ^1^ Psychology Department, Psychophysiology Lab, University of Arizona, Tucson, AZ, United States; ^2^ Openwater, San Francisco, CA, United States

**Keywords:** mood disorder, transcranial ultrasonic neuromodulation, repetitive negative thinking (RNT), depression, default mode network

## Abstract

**Introduction:**

Up to 50% of individuals fail to respond to current depression treatments. Repetitive negative thought and default mode network hyperconnectivity are central in depression and can potentially be targeted using novel neuromodulation techniques. This community-based study assessed whether a treatment using non-invasive transcranial focused ultrasound targeting the default mode network can decrease depression symptoms and repetitive negative thought, and improve quality of life.

**Methods:**

Study recruitment began in August 2023 and ended in February 2024. Twenty individuals aged 18 – 50 were enrolled from among 247 screened. Exclusion criteria included history of psychosis/mania, acute suicidality, MRI contraindications, pregnancy, and medical and neurological factors that may complicate diagnosis or brain function. Participants completed up to three weeks of transcranial ultrasound (11 sessions) targeting the anterior medial prefrontal cortex; ten minutes per session. Depression severity (Beck Depression Inventory – II and the Hamilton Depression Rating Scale), repetitive negative thought (Perseverative Thinking Questionnaire), and quality of life (World Health Organization Quality of Life Scale) were outcomes.

**Results:**

This sample was young (mean 30.4 years ± 10.0), predominantly female (75%), with moderate to severe depression and high comorbidity. Fifty percent of participants endorsed current psychiatric medication use. Ten percent of subjects dropped out of the study due to time constraints. Significant decreases in depression were observed over the course of treatment on self-report, 10.9 (p < 0.001, CI = -13.55, -7.92) and interview depression ratings, 4.2 (p < 0.001, CI = -5.85, -2.62), as well as significant decreases in repetitive negative thought, 8.4 (p <0.001, CI = -10.55, -6.03). Improvements in physical and psychological well-being were also observed over the course of treatment, 7.2 (p < 0.001, CI = 3.64, 10.63) and 11.2 (p < 0.001, CI = 7.79, 14.49), respectively, as well as improvements in environment satisfaction, 5.0 (p =0.001, CI = 2.24, 7.56).

**Discussion:**

Non-invasive transcranial focused ultrasound holds promise as a treatment for depression holds promise as a treatment for depression, however, future work including control arms is required to ascertain its causal role in depression.

**Clinical trial registration:**

https://clinicaltrials.gov/study/NCT06320028intr=Ultrasound&cond=depression&locStr=Arizona&country=United%20States&state=Arizona&rank=1, identifier NCT06320028.

## Introduction

Depression is a leading cause of disability (1), affecting 21 million adults and significantly diminishing quality of life (2). Major Depressive Disorder (MDD) is typically recurrent (3–5), and impairment is compounded with subsequent episodes (6). Critically, current interventions are not effective for certain profiles of depression (7, 8).

In conjunction with depressed mood and related symptoms, Repetitive Negative Thought (RNT) has been identified as a maintaining factor in depression (9), as well as a predictor of depression improvements (8). The brain’s Default Mode Network (DMN), which has greater connectivity during self-referential processing [e.g., mind-wandering (10, 11)] and, in particular, *negative* self-referential processing [e.g., RNT (12)], is also shown to play an important role in depression. Studies have identified that greater DMN connectivity (e.g., hyperconnectivity) has been associated with greater depression severity and RNT (13, 14). Together, these findings highlight the mechanistic roles that RNT and DMN hyperconnectivity play in the development and maintenance of depression.

Because roughly 50% of depressed individuals are treatment-resistant to traditional treatments (7, 15), more effective interventions are needed, ideally those deriving from a better mechanistic understanding of depression. DMN connectivity has been altered (e.g., using transcranial magnetic stimulation (TMS), psychedelics, meditation) in various clinical populations (16, 17), with the goal of improving treatment approaches. A novel neuromodulation technique, non-invasive Transcranial Focused Ultrasound Stimulation (tFUS), holds promise in the treatment of depression (18, 19).

Unlike other noninvasive methods (TMS and transcranial electrical stimulation (TES) using direct (tDCS) or alternating (tACS) current), tFUS uses low-intensity ultrasound involving a focused nonthermal ultrasound beam, which safely passes through the skull (20) to exert electro-mechanical effects on target neurons, including the ability to induce excitatory and inhibitory effects depending on the ultrasound parameters used (21, 22). tFUS also presents advantages beyond other non-invasive neuromodulation techniques (e.g., TMS) due to its ability to target deeper brain regions with greater precision (22), without side effects (e.g., skin irritation, local pain) that can accompany techniques like TMS (23).

Limited research supports tFUS as a treatment for depression. Resnik and colleagues examined tFUS targeting the right inferior frontal gyrus, a component of the executive control network, on symptoms of depression; those engaging in a five-day treatment regime experienced a decrease in worry (18) compared to those receiving sham. Additionally, Sanguinetti and colleagues also found that tFUS decreased negatively-valanced emotions and altered DMN connectivity (19). These findings provide the foundation for further exploring the use of tFUS as a treatment for depression.

The present study aimed to assess whether treatment using tFUS delivered to the anterior medial prefrontal cortex (amPFC), a hub of the DMN (11), can decrease depression symptoms and RNT, improve quality of life, and whether changes in depression severity are mirrored by changes in RNT.

## Methods

The Institutional Review Board of the University of Arizona approved the experimental protocol (IRB approval number: STUDY00002019). All participants signed an informed consent document before participation. Participants were recruited from August 2023 to February 2024.

Clinical Trial Registration number: 019782-00001, https://clinicaltrials.gov/study/NCT06320028intr=Ultrasound&cond=depression&locStr=Arizona&country=United%20States&state=Arizona&rank=1 identifier, NCT06320028.

### Participants

Individuals with a current major depressive episode, assessed using the Structured Clinical Interview for the DSM-5 (SCID-5) (24) were enrolled. They also experienced clinically significant RNT, characterized by a total score on the Perseverative Thinking Questionnaire (PTQ) (25) above the 75% percentile (≥37).

The SCID-5 is a gold-standard, semi-structured clinical interview tool used to assess psychiatric disorders recognized by the Diagnostic and Statistical Manual of Mental Disorders, 5^th^ edition (DSM-5) (26), including modules assessing current episode and history of depression, mania and psychosis, substance-use, anxiety-related disorders, and posttraumatic stress (24). The interrater reliability of the SCID-5 has been extensively validated, with published kappa coefficients ranging from 0.66 to 0.83 across various diagnostic modules (24), indicating good agreement on categorical judgements between raters. The PTQ is a self-report measure consisting of 15-items measuring the degree of negative thinking patterns (e.g., The same thought keeps going through my mind, Thoughts intrude into my mind) using a Likert scale of 0 (never) to 4 (almost always) for each question (25). Validation studies indicate that PTQ is a highly reliable measure of RNT (α =0.95) (25).

Participants were ages 18 – 50, right-handed, English-speaking, and without any neurological symptoms or symptoms of mania/psychosis. Additional exclusion criteria included: history of head injury with loss of consciousness; uncorrected vision and/or hearing impairment that would interfere with study participation; current or history of brain or mental illness judged likely to interfere with testing, including drug and/or alcohol dependence; a diagnosed sleep disorder (e.g., Insomnia); current drug, alcohol or prescription drug intoxication; history of epilepsy; history of diagnosed migraines; metal implants in head or face, including permanent dental retainers; history of cardiac problems that could impact brain function (e.g., atrial fibrillation); and current active suicidal ideation necessitating immediate treatment. During the consent process, participants were instructed to maintain their current medication and psychotherapy regimens and not make any changes for the duration of their study participation.

### Overview of ultrasound treatment protocol

Eligible participants completed up to three weeks of ultrasound treatment. Before treatment, they completed an MRI session, a clinical interview, and self-report surveys. The first week of ultrasound involved five sessions within a seven-day period. Participants completed the same baseline assessments after completing week 1, and if they did not meet early remission criteria (defined below), they continued tFUS treatment for for two more weeks, three sessions per week, each within a seven-day period. Participants completed the same series of assessments after week 3. Participants completed a subset of the symptom outcome measures after completing week 1 and week 3 (weekly), and some after each tFUS session (daily).

### Symptom outcome measures and adverse event tracking

Before any ultrasound intervention sessions, participants completed baseline surveys: Beck Depression Inventory-II (BDI-II) (27), PTQ (25), Hamilton Depression Rating Scale (HDRS) (28), the World Health Organization Quality of Life Scale (WHOQOL-BREF) (29), and the Columbia Suicide Severity Rating Scale (CSSRS) (30).

The BDI-II is a self-report measure consisting of 21 items measuring current, key symptoms of depression (e.g., sadness, loss of interest, suicidality) using a Likert response scale from 0 to 4 (e.g., 0 – I do not feel sad; 4 – I am so unhappy I cannot stand it) (27). The Hamilton Depression Rating Scale (HDRS) is a 17-item interview administered by a clinician to assess current key depression symptoms (e.g., depressed mood, pathological guilt, Suicide) on a Likert scale of 0 to 4 (e.g., 0 – absent; 4 – severe: Patient reports virtually only these feeling states in verbal and non-verbal communication, *or* depressed almost every day and missed three or more days of work *or* reports suicidal ideation for three or more days) (28). Both the BDI-II and HDRS have excellent published reliability [BDI-II α = 0.93 (27) and HDRS interrater reliability = 0.90 (28)]. As previously mentioned, the PTQ is a highly reliable measure of RNT (α =0.95) (25).

The WHOQOL-BREF is a 26-item self-report measure assessing four aspects of quality of life (QOL): physical well-being, psychological well-being, social satisfaction, and environment satisfaction (29). This measure uses a Likert scale of 1 to 5 for each question (e.g., How would you rate your quality of life? 1 – very poor; 5 – very good). The WHOQOL-BREF is a reliable measure of QOL with published alpha coefficients ranging from 0.66 – 0.8 across the four domains of QOL (29).

The CSSRS is an assessment tool for evaluating the severity of suicidal ideation and behaviors, measuring key aspects such as the intensity and frequency of suicidal thoughts, associated intent, and types of behaviors (e.g., actual, aborted, or interrupted attempts) (30). It includes both “yes or no” questions (e.g., “Have you wished you were dead or wished you could go to sleep and not wake up?”) and scaled questions (e.g., "When you have the thoughts how long do they last?": 1 - easily able to control thoughts; 5 - more than 8 hours/persistent or continuous). In prior work, the CSSRS demonstrates 100% sensitivity and specificity for identifying actual and interrupted attempts, and 99.4% specificity and 100% sensitivity for identifying aborted attempts, demonstrating high accuracy in identification while minimizing false positives (30). This measure was used in the present study to track changes in suicidal ideation throughout treatment.

These measures were re-administered following the conclusion of treatment after 1 week and 3 weeks (if applicable) of ultrasound sessions to assess weekly changes in symptoms. In addition to being administered before and after treatment, the BDI-II and PTQ were administered after each ultrasound session to assess daily symptom progression.

Before each ultrasound session, subjects were asked whether they experienced adverse events that may be due to the ultrasound. For reported events, the onset and duration of the event were noted, the severity was rated, and the relationship to study procedure was assessed. After each ultrasound session, participants completed a sensation questionnaire to assess sensations subjects may have experienced from the ultrasound, including: itching, heat/burning, tingling, vibrating/pulsing, sound, tension, and pain. Before beginning each subsequent ultrasound session (e.g., at the beginning of the next session) and acutely after completion of the sensation questionnaire, subjects were asked whether they experienced any sensations or other issues during the ultrasound session. For reported events, further probing would determine whether an adverse event related to the study occurred. If related to the study, the onset and duration of the event were noted, the severity rated, and the relationship to study procedure assessed. Additionally, SWI MRI images were collected at baseline and after treatment conclusion to provide an objective index of whether ultrasound may have created any damage to neurons or vasculature (see MRI scans section for more detail).

#### Early remission, remission, and response criteria

To meet early remission criteria following week 1, participants must have a BDI-II score of < 13 and a HDRS score of < 8, and a PTQ score of < 18. If any of these criteria were not met, the participant continued treatment for two additional weeks.

After completion of the treatment protocol (i.e., after week 1 or week 3), remission (defined above) and response were assessed, with a decrease of scores below 50% of baseline considered a response as commonly used in previous treatment literature (15, 31).

### MRI scans

Scanning sessions included a T1 weighted structural scan, PETRA short TE scan (skull density), twelve-minute BOLD functional resting-state scan, and Susceptibility Weighted Image (SWI) before beginning ultrasound treatment, after one week of treatment, and after three weeks of treatment (if applicable). The PETRA scans were used for localization and targeting and the SWI images were assessed by board-certified neurologists to assess micro-hemorrhaging. Other MRI acquisitions are not analyzed here and will be reported in a separate paper.

### Ultrasound session procedures, device specifications, and targeting precision

After localization and placement of the ultrasound device, each ultrasound session took ten minutes to complete. Participants were instructed to sit quietly, keeping their eyes open. After the ultrasound treatment was complete, the participant sat quietly for another 20 minutes, with eyes open or closed and letting their thoughts come and go.

tFUS was delivered using a custom Neuromodulation device (32) consisting of 128 element ultrasound array (Openwater) with the steerable ultrasound beam having the following parameters: acoustic frequency = 400 kHz, pulse duration = 5 ms, pulse repetition rate (PRR) = 10 Hz, a maximal spatial peak/temporal average acoustic intensity = 670 mW/cm^2^, peak negative pressure 820 kPa. The ultrasound probe was secured by a custom-designed headset created by Openwater. Localite Neuronavigation Software (TMS Navigator 3.3 adapted for ultrasound device) and hardware registered the position of the probe with respect to the patient’s structural MRI, providing information to develop a novel electronically-steered, stereotactic tFUS treatment plan to the personalized target for each participant’s left anterior-medial prefrontal cortex [amPFC; MNI Coordinates -5, 45, -3 (10, 33, 34)]. This target was selected because this region was defined by resting-state connectivity, showing high between-node centrality as a DMN hub and showing a large main effect of self-relevancy in task-related paradigms (10).

**Figure 1:**
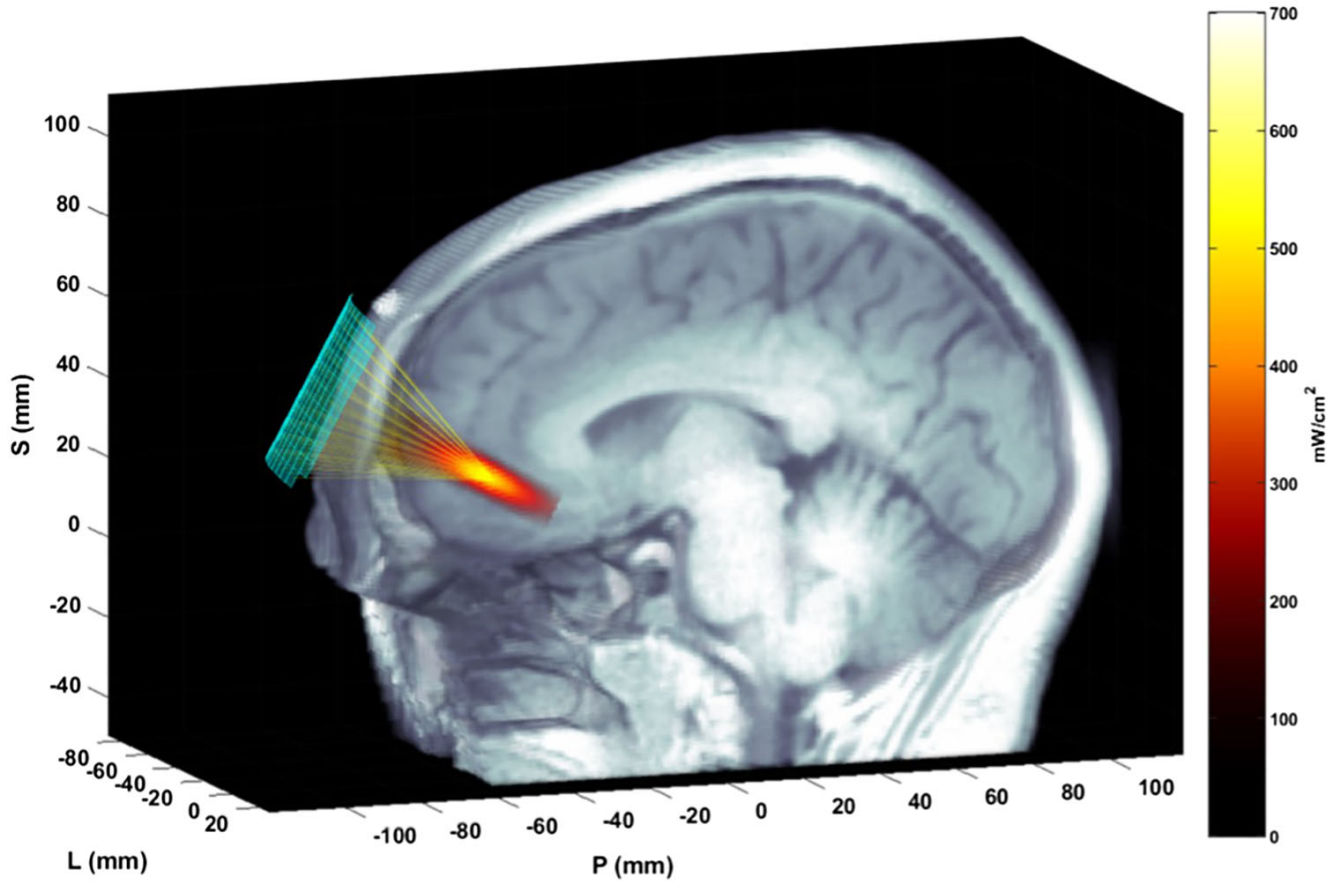
Ultrasound focusing to the amPFC. The matrix array transducer is positioned on the forehead and focuses sound through the skull and to the target. The transducer position is measured with the Localite TMSNavigator Neuronavigation system (Localite GmbH, Bonn, Germany). A focal spot, modeled based on the computed time delays using the ultrasound simulation package K-Wave, is overlain on the MRI image, representing the pulse-averaged spatial distribution of applied acoustic intensity.

The ultrasound array in the custom headset was affixed at the general location of the amPFC target (MNI coordinates: -5, 45, -3) with precise targeting achieved by electronic steering within limits that meet safety parameters for ultrasound exposure (32) (**Figure 1**). A multi-foci, radial pattern approach was used that distributed the delivered energy in five sub-foci within 5mm from each other (which is the width of the focus in the nominal place, as defined by the -6dB pressure region). The K-Wave modeled peak energy delivery relative to the target location was highly accurate, with the -3dB centroid location of the focus falling within 1.0 +/- 1.1mm of the data measured with a hydrophone in a water tank (.02 +/-.276 mm in the lateral-axial plane, and.87 +/- 1.2mm in the axial direction). The actual pressure values estimated in K-wave and measured in the water tank agreed within 3.6 +- 1.2% within the -6dB contours. For a detailed description of translating MNI coordinates of our target into participant native space, as well as more information about the modeling approach, please see Bawiec et al. (2024) (32).

### Statistical analysis

For all statistical analyses, an alpha of 0.05 was employed and significance tests were two-tailed. Analyses were conducted in R studio (version: 2023.09.1 + 494) (35).

Seven Multi-level Models (MLM), which can account for missing data and within-subject variability, were used to assess change in the main outcomes of interest: depression symptoms (BDI-II and HDRS), RNT (PTQ), and four subscales reflecting QOL (WHOQOL-BREF physical well-being, psychological well-being, social satisfaction, and environment satisfaction subscales).

In each model, “time” was specified as the independent variable, modeling the average change in symptoms across timepoints. A random intercept was specified to account for within-subject variation in baseline symptoms.

Full information maximum likelihood estimation was applied to each model to handle missing data from three subjects who did not complete post 3 assessments. A Satterthwaite degrees of freedom adjustment was applied to each model to account for the small sample size.

Given that “time” was already scaled from 0 to 2 (baseline = 0; week 1 = 1; week 3 = 2) centering was not required. This scaling represents the progression of assessment timepoints. Bootstrap confidence intervals (CIs), a non-parametric approach that resamples the data to estimate the distribution of the model parameters, were used to assess the robustness of the results. Bootstrapping is ideal for small sample sizes and data with considerable variability.

Two linear regression models assessed the relationship between change in depression symptoms and change in RNT. Model one assessed the relationship between change in self-report depression symptoms (BDI-II) and change in RNT (PTQ) and model two assessed the relationship between change in clinical interview depression ratings (HDRS) and change in RNT (PTQ). Change scores for the BDI-II, HDRS, and PTQ were calculated as baseline minus post, with greater change values indicating a greater decrease in depression symptoms and RNT.

## Results

### Sample characteristics

**Figure 2:**
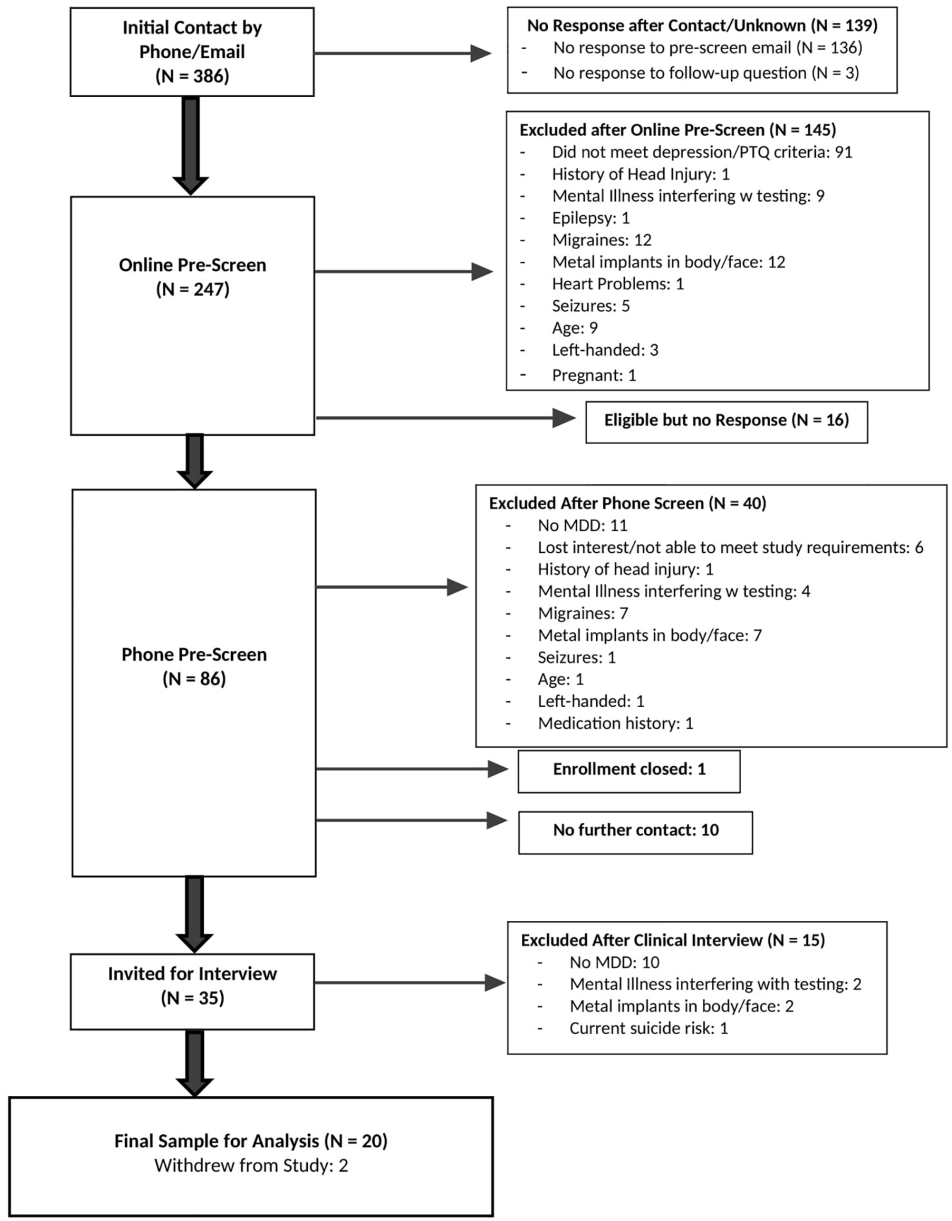
CONSORT diagram. Diagram showing participant flow through the study procedures.

From among 386 individuals initially contacted, 247 completed the initial pre-screen web-based survey. Eighty-six potential participants completed a phone screen to confirm responses on the pre-screen survey related to eligibility, and 35 completed the SCID for DSM-5 to confirm a diagnosis of current depression and an absence of mania/psychosis. Twenty participants were enrolled in the study (CONSORT diagram in **Figure 2**). Participant demographics are presented in **Table 1**. This relatively young (mean 30.4 years ± 10.0) and predominantly female (75%) sample had moderate to severe depression (BDI-II = 38.9 ± 9.3, HDRS = 19.9 ± 6.3, PTQ = 144.4 ± 6.2). The sample was also highly comorbid, and more than half had early onset depression (before the age of 13). Fifty percent of participants were currently taking medication related to their anxiety and/or depression during the intervention.

**Table 1:** Participant demographics.

Demographics		N = 20
Age, Mean (SD)		30.35 (10.04)
Gender (F/M/Other), No. %		75/20/5
Years of education, Mean (SD)		13.83 (1.93)
Race, No. %		
	White	45
	Black	10
	Chinese	5
	Middle Eastern	5
	Indian	5
	Unknown	30
Ethnicity, No. %		
	Hispanic	0
	Non-Hispanic	70
	Unknown	30
Employment, No. %		
	Full-time	15
	Student	15
	Part-time	45
	Unemployed	25
Baseline BDI-II, Mean (SD)		38.85 (9.34)
Baseline PTQ, Mean (SD)		44.35 (6.24)
Baseline HDRS, Mean (SD)		19.90 (6.34)
Depression onset (Early/Teen/Adult), No. %		55/25/20
Comorbidities, No. %		
	Anxiety and Stress-related Disorder	85
	Trauma-related Disorder	15
	Attention Deficit Hyperactivity Disorder	35
	Eating Disorder	5
	Persistent Depressive Disorder	55
History of Suicidal Ideation (Passive/Active/None), No. %		30/60/10
Hospitalization History (Any), No. %		35
History of Suicide Attempts (None/One/Multiple), No. %		70/15/15
Current Treatment (Medication/Psychotherapy/None), No. %		50/20/10
Past Treatment (Medication/Psychotherapy/None), No. %		75/60/10
Current Medication Type, No. %		
	SSRI (Luxov, Prozac, Sertraline)	15
	SARI (Trazadone)	5
	NDRI (Wellbutrin)	10
	Anti-convulsant (Gabapentin, Lamotrigine)	15
	Beta-Blockers (Propranolol)	5
	CNS stimulant (Adderall, Vyvanse)	10
	Sedative (propofol)	5
	Anti-hypertensives (Clonidine)	10

Thirty percent of participants did not provide race and ethnicity information. For the 70% of participants that did complete demographic information, 45% of participants identified as White, 10% Black, 5% Chinese, 5% Middle Eastern, and 5% Indian. Seventy percent of participants identified as non-Hispanic. Additionally, 45% of participants were employed part-time and 15% employed part-time, 15% were students, and 25% of participants were unemployed at the time of study enrollment.

### Adverse events

Dropout rate, as one index of the acceptability of tFUS treatment, was low: 10% (2/10) did not complete treatment, discontinuing after week 1 of treatment due to lack of symptom improvement. Dropout was not due to adverse events.

No serious adverse events were reported. Reported sensations (itching, heat/burning, tingling, vibrating/pulsing, sound, tension, and pain) are presented in **Table 2**; for aversive sensations, the modal and median endorsement was 0 (no sensation). All means were below 2.2 on the 10-point scale. For pain and tension specifically, individual reports attributed the pain and tension to the tightness of the headset, not the ultrasound itself. Additionally, none of the participants endorsed suicidal ideation posing imminent risk to self. One subject reported a transient increase, compared to baseline, in suicidal ideation during the post 3 assessment due to a “relationship breakup” unrelated to study procedures.

**Table 2:** Sensation intensities reported on the sensation questionnaire.

Sensation	Mode	Median	Mean	Std Dev	Min	Max
Pain	0	0	0.91	1.76	0	7
Itching	0	0	0.28	0.82	0	7
Heat/Burning	0	0	0.65	1.14	0	5
Tingling	0	1	0.87	1.62	0	8
Vibrating/Pulsing	0	0	1.20	1.64	0	8
Sound	0	0	1.36	1.92	0	10
Tension	0	0	1.63	2.16	0	8

SWI images acquired at baseline before tFUS sessions and again after week 1 and week 3 were read by two board-certified neuroradiologists. SWI images are sensitive to vascular micro-hemorrhages. All 20 scans per timepoint were determined to be normal with no findings on SWI, indicating that there were no microhemorrhages resulting from tFUS delivery. Three participants’ baseline SWI readings revealed nonspecific white matter hyperintensities which may be seen with chronic microangiopathic ischemic changes and decreased susceptibility which may be related to microhemorrhages. With no change in the pre and post treatment MRI scans of these presumed microhemorrhages, they were deemed chronic.

### Depression symptoms and RNT

**Figure 3:**
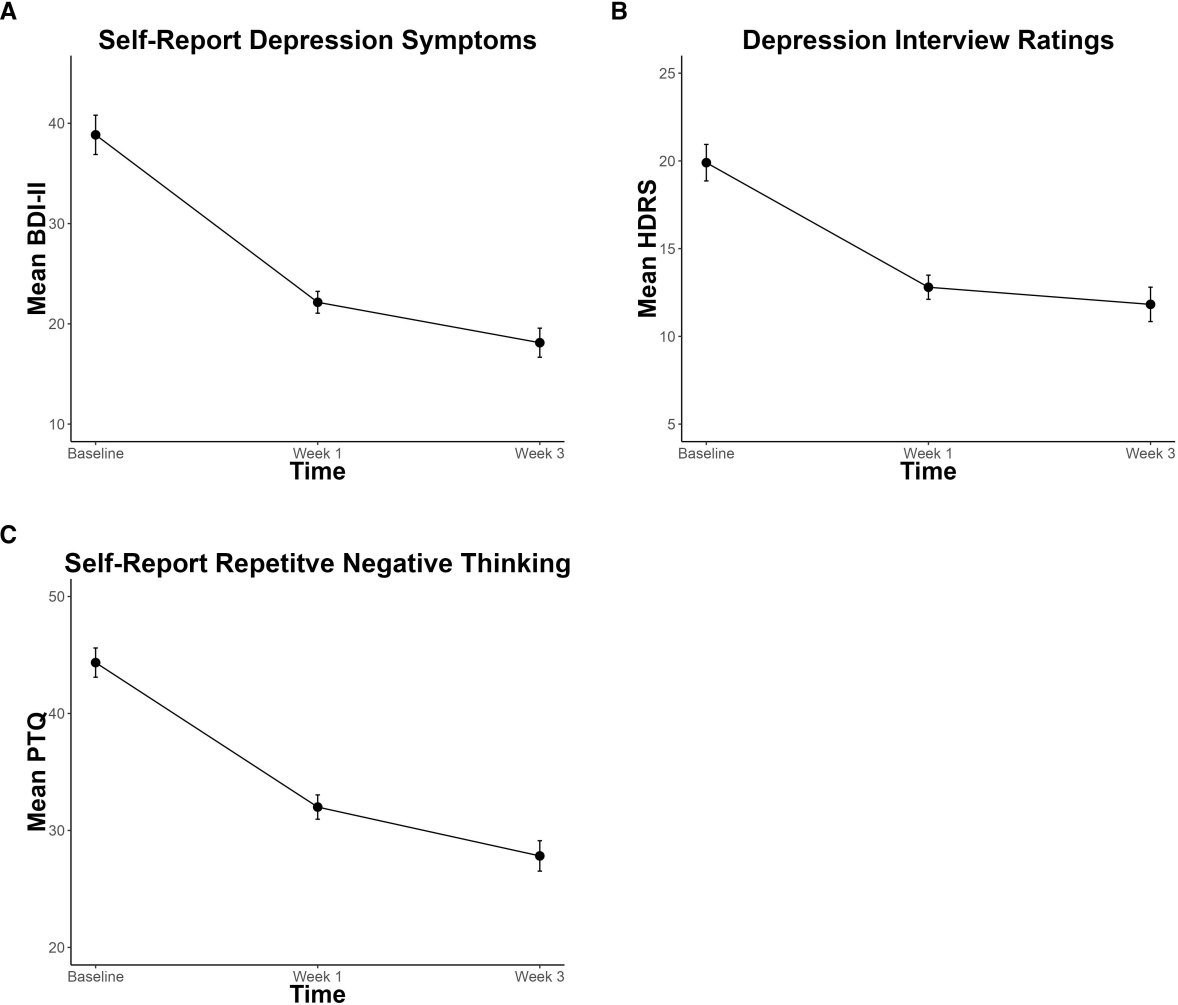
Significant decreases in depression symptoms and repetitive negative thought over the course of non-invasive Transcranial Focused Ultrasound Treatment, assessed by **(A)** Beck-Depression Inventory – II (BDI-II), **(B)** Hamilton Depression Rating Scale (HDRS), and **(C)** Perseverative Thinking Questionnaire (PTQ). Error bars represent within-participant standard error.

For the BDI-II and HDRS, respectively, 60% and 45% of all 20 participants met response criteria. Thirty-five percent (7/20) met remission criteria for both the BDI-II and HDRS. Significant decreases in depression severity and RNT were observed (**Figure 3**). Depression symptoms, characterized by the BDI-II and HDRS total scores, significantly decreased by 10.9 (p < 0.001, CI = -13.55, -7.92) and 4.2 (p < 0.001, CI = -5.85, -2.62), respectively, across time. RNT, characterized by PTQ total scores, also significantly decreased by 8.4 (p <0.001, CI = -10.55, -6.03), across time.

**Figure 4:**
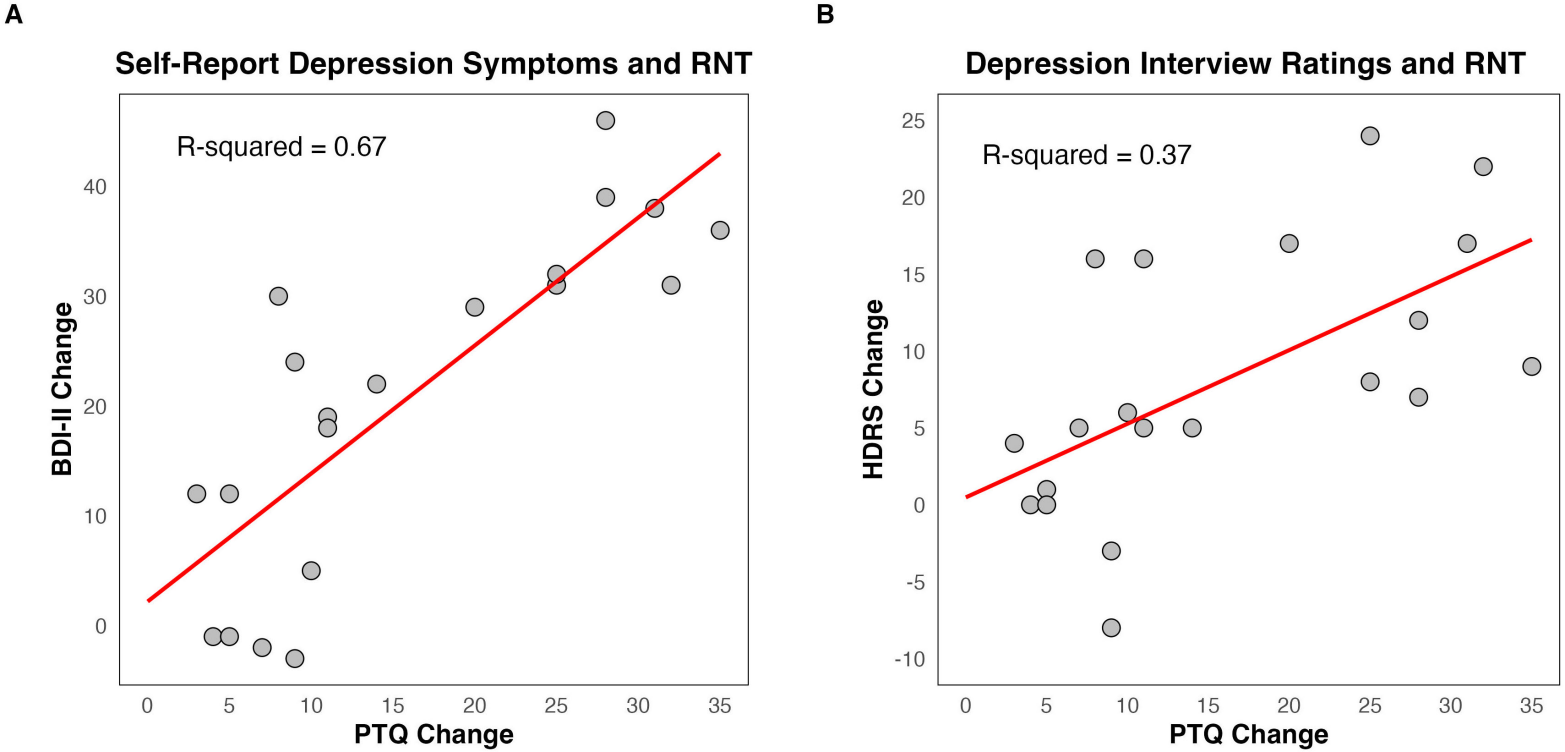
Relationship between change in depression symptoms and change in repetitive negative thought (RNT). **(A)** Change in Beck Depression Inventory – II (BDI-II) and change in Perseverative Thinking Questionnaire (PTQ). **(B)** Change in Hamilton Depression Rating Scale (HDRS) and change in PTQ. The scatter plot represents a linear regression containing the R-squared value to assess the strength of the relationship and the red line to visualize the linear fit. Change scores for BDI-II, HDRS, and PTQ were computed as baseline minus post scores, meaning greater positive numbers reflect a greater decrease in depression symptoms and RNT.

There was a significant positive relationship between change in depression and change in RNT (**Figure 4**), for both the BDI-II self-report (*R* ^2^ = 0.67, F = 36.84 (1, 18), p < 0.001, CI = 0.76, 1.57) and HDRS interview ratings (*R* ^2^ = 0.37, F =10.59 (1, 18), p = 0.004, CI = 0.17, 0.79).

### Quality of life

**Figure 5:**
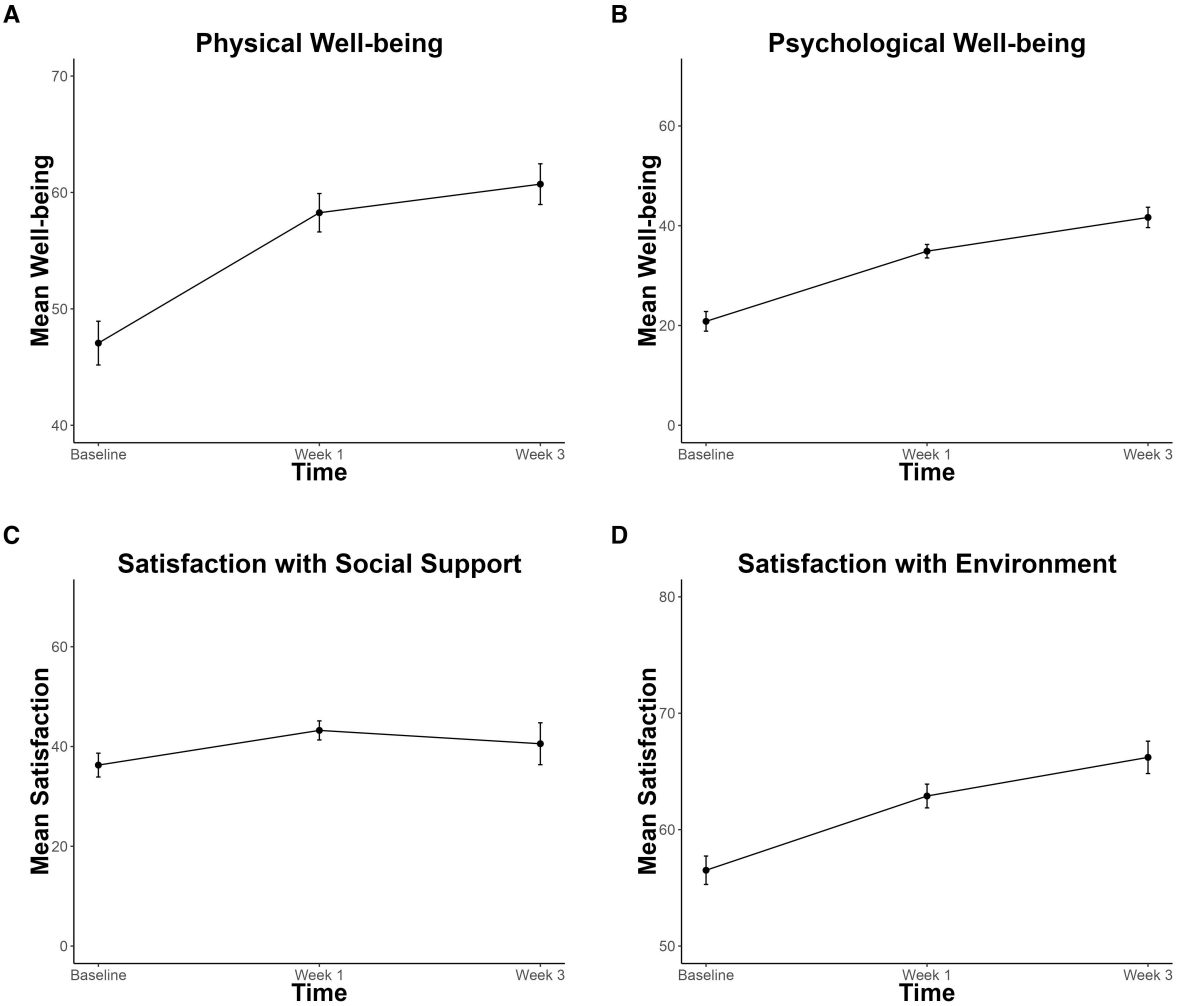
Improvements in Quality of Life. **(A)** Physical Well-being **(B)** Psychological Well-being **(C)** Social Satisfaction **(D)** Environment Satisfaction subscales of the World Health Organization Quality of Life Scale (WHOQOL-BREF). Significant improvement is found for panels **(A, B, D)**. Error bars represent within-participant standard error.

Physical and psychological well-being significantly improved by 7.2 (p < 0.001, CI = 3.64, 10.63) and 11.2 (p < 0.001, CI = 7.79, 14.49) and environment satisfaction improved by 5.0 (p < 0.001, CI = 2.24, 7.56), across time (**Figure 5**). No significant improvements in social satisfaction were observed (p = 0.15, CI = -0.87, 6.61).

## Discussion

### Adverse events

Transcranial focused ultrasound treatment for depression using a novel, electronically-steered, stereotactic approach was successfully delivered without serious adverse events. Participants only reported transient, mild to moderate discomfort (e.g., tension and pain) which is similar to sensations experienced in many neuromodulation treatments for depression, such as rTMS (36). Unlike TMS or tDCS, where the source of the pain and discomfort is largely due to the delivery of the magnetic stimulation itself (e.g., skin irritation, local pain) (23), several participants identified the source of the pain and tension to be from the headset. Unlike other neuromodulation techniques, such as TMS, where up to 22.6% of participants experienced headaches from the active treatment (37), there were no reports of headaches related to tFUS delivery.

On average, previous neuromodulation techniques experience a 4.5% dropout rate due to stimulation-related adverse events (38, 39). In the present study, zero percent of participants dropped out due to tFUS-related adverse events and only 10% of participants dropped out due to lack of positive effects of the treatment, which is also significantly better than dropout rates in traditional clinical depression trials, such as individual psychotherapy and pharmaceuticals with up to one-third drop out prior to treatment completion (40–42). Overall, these findings support the notion that not only is tFUS comparably safe to novel interventions such as TMS and tDCS, it may also have fewer side effects and lower dropout compared to other neuromodulation techniques.

### Decreases in depression symptoms and RNT

There was a significant, observed decrease in depressed mood and RNT in individuals with current major depression over the course of treatment in just three weeks. For the BDI-II and HDRS, respectively, 60% and 45% of participants met response criteria. These percentages are comparable to traditional treatments for depression, such as antidepressants and psychotherapy (45 – 55%) in samples without substantial comorbidity; (43). The rates in the current study were achieved despite substantial comorbidity, a known poor prognostic sign (44).

A potential advantage of tFUS compared to traditional interventions is the rapidity of response: the response rate of 45-60% and remission rate of 35% occurred after just three weeks of treatment, which exceeds what has been found in rTMS interventions for depression with remission rates of as little as 18.6% and up to 30% after up to six weeks of treatment involving more sessions (36, 45). The response from tFUS also occurred with fewer sessions (11) than traditional cognitive behavioral therapy (46) [~12 – 20 sessions, once or twice per week (47)]. Given the open-label design without a control group, it is not possible to infer that tFUS is the causal reason participants experienced decreases in depression symptoms and RNT at this time. This study, however, shows initial promise for the application of tFUS for treating MDD with the potential to offer a more rapid response than traditional treatments.

### Improvements in quality of life

Physical and psychological functioning, as well as satisfaction with one’s environment, significantly increased over the course of treatment. This extends previous clinical intervention work where quality of life is not commonly considered a main outcome in treatments for depression (48, 49). Additionally, certain treatments (e.g., antidepressants) fail to lead to greater improvements in quality of life compared to controls (50), which prompts an important re-evaluation of what “improvement” means when developing and validating treatment protocols. It will be critical in future work to assess sustained changes in quality of life resulting from tFUS for depression, as well as treatments for depression generally.

The lack of improvement in social satisfaction after tFUS suggests the potential for future tFUS studies to augment tFUS with interventions that are known to improve social relationships and support, such as interpersonal psychotherapy and cognitive behavioral therapy (51), as a multimodal package that addresses the full dimensionality of improving QOL. Despite the promise of tFUS on quality of life in depressed individuals based on these findings, future work with control arms is needed to ascertain the causal role of tFUS in depression.

### Impact of tFUS on the DMN

tFUS is a novel neuromodulation technique that holds promise as a tool that can directly modulate brain function with precision (22). Although the direct immediate impact of tFUS on functional connectivity was not assessed in the present study, it is hypothesized that the tFUS parameters used in this open-label case series (pulse repetition rate = 10Hz, acoustic frequency = 400kHz) promoted an inhibitory effect on brain connectivity. Low pulse repetition frequency of tFUS coupled with lower acoustic frequency have been shown to have an inhibitory effect on brain activity by weakening neural firing patterns (52–55). Lord and colleagues demonstrated that targeting the other major hub of the DMN, the posterior cingulate cortex (PCC), using similar inhibitory tFUS parameters (pulse repetition frequency = 10.526Hz, acoustic frequency = 500kHz) in a healthy sample had an inhibitory effect on DMN connectivity, where there was observed decrease in connectivity between the amPFC and PCC (56). The precise mechanism of how the delivery of ultrasound energy translates to changes in neural activity, however, remains a matter of some debate (21), and more research is needed to confirm its inhibitory effects on neural function.

### Role of RNT and the DMN in depression

There was a significant, positive relationship between the change in depression symptoms and change in RNT, wherein those with greater decreases in RNT experienced greater decreases in depression symptoms. These findings support previous literature identifying the relationship between RNT and depression (8, 9), however, future work requiring larger sample sizes and a control group should aim to apply more sophisticated models coupled with longitudinal datasets to assess a predictive relationship between RNT and depression.

Our results also provide preliminary support regarding the DMN’s role in depression and RNT, as we were successfully able to decrease symptoms while directly targeting a major hub of the DMN. Although the casual relationship between DMN connectivity, depression symptoms, and RNT was not assessed in the present study, it is hypothesized that through directly inhibiting DMN function, resulting in a decrease in functional connectivity within the DMN, participants are experiencing decreases in RNT and depression symptoms. It is critical that future research, namely randomized clinical trials, aim to assess the causal relationship between changes in DMN connectivity, RNT, and depression symptoms, as well as the temporal relationship between change in RNT and change in DMN connectivity throughout the course of tFUS treatment. Further evidence will include resting-state functional connectivity MRI analysis to assess whether changes in DMN connectivity track changes in depression symptoms and RNT.

### Limitations and future directions

The present study provides important, preliminary evidence for the potential use of tFUS as a novel, targeted intervention for depression. A critical limitation is that this study was an open-label unblinded trial with a relatively small sample size and, as such, the present study was not able to assess the causal role of tFUS targeting the amPFC in depression treatment. To assess whether there is a causal relationship between tFUS delivery and a decrease in depression symptoms and RNT, a randomized controlled trial with active and sham ultrasound is needed to control for nonspecific factors and minimize the impact of a placebo effect.

Limitations related to the delivery of tFUS include choosing a target (amPFC) that requires traversing a region with thicker skull density compared to other potential DMN targets (e.g., PCC) and, as a result, delivering less energy to the target due to dispersion of the tFUS signal. However, we are confident that some energy was delivered, and although we cannot infer causality without a control group, we also observed decreases in depression symptoms in the present study. It is, therefore, unclear whether targeting the amPFC is the most potent approach for modulating DMN connectivity in relation to decreasing depression symptoms, and future work incorporating control arms is needed to dissect the differential impact of targeting different hubs of the DMN (56). An empirical question that also still remains is whether engaging in tasks or activities acutely after ultrasound delivery amplify or attenuate effects (57). Future work should aim to understand the optimal protocol for neuromodulation delivery (TMS, TDCS, tFUS). Finally, future work should employ cognitive measures that may relate to symptom improvement and DMN targeting (58–60). Despite these limitations, the present findings provide a strong foundation for the implementation of tFUS as a treatment for depression with pronounced and rapid observed anti-depressant effects over the course of treatment, suggesting the promise of a randomized clinical trial.

## Data Availability

The raw data supporting the conclusions of this article will be made available by the authors, without undue reservation.
